# Whole-Genome Sequencing Redefines *Shewanella* Taxonomy

**DOI:** 10.3389/fmicb.2019.01861

**Published:** 2019-08-28

**Authors:** Kaisa Thorell, Jan P. Meier-Kolthoff, Åsa Sjöling, Alberto J. Martín-Rodríguez

**Affiliations:** ^1^Department of Infectious Diseases, Institute of Biomedicine, Sahlgrenska Academy, University of Gothenburg, Gothenburg, Sweden; ^2^Department of Bioinformatics, Leibniz Institute DSMZ-German Collection of Microorganisms and Cell Cultures GmbH, Brunswick, Germany; ^3^Centre for Translational Microbiome Research, Department of Microbiology, Tumor and Cell Biology, Karolinska Institutet, Stockholm, Sweden

**Keywords:** phylogenomics, GBDP, digital DDH, species delineation, taxonomic revision, comparative genomics

## Abstract

The genus *Shewanella* encompasses a diverse group of Gram negative, primarily aquatic bacteria with a remarkable ecological relevance, an outstanding set of metabolic features and an emergent clinical importance. The rapid expansion of the genus over the 2000 s has prompted questions on the real taxonomic position of some isolates and species. Recent work by us and others identified inconsistencies in the existing species classification. In this study we aimed to clarify such issues across the entire genus, making use of the genomic information publicly available worldwide. Phylogenomic analyses, including comparisons based on genome-wide identity indexes (digital DNA-DNA hybridization and Average Nucleotide Identity) combined with core and accessory genome content evaluation suggested that the taxonomic position of 64 of the 131 analyzed strains should be revisited. Based on the genomic information currently available, emended descriptions for some *Shewanella* species are proposed. Our study establishes for the first time a whole-genome based phylogeny for *Shewanella* spp. including a classification at the subspecific level.

## Introduction

The family *Shewanellaceae*, was formally established by Ivanova et al. (2004) 15 years ago. The type (and only) genus within this family is *Shewanella*, which had been defined already by MacDonell and Colwell after their taxonomic revision of the family Vibrionaceae (MacDonell and Colwell, 1985) and named after J. M. Shewan in recognition for his work in the microbiology of fish and fishery products (Shewan et al., 1960). *Shewanella* are Gram negative, facultative anaerobic, rod-shaped bacteria with a single polar flagellum, most of which are able to grow at low temperature. Some species produce polyunsaturated fatty acids. The type species is *Shewanella putrefaciens* (MacDonell and Colwell, 1985).

*Shewanella* are ubiquitously distributed in marine and freshwater environments, including deep-sea and polar regions, with some species being part of the microbiota of aquatic animals. One species, *S. algae*, is considered to be an emergent human pathogen (Martín-Rodríguez et al., 2017), with a few additional species being occasionally pathogenic. While human infections are still relatively scarce, the number of case reports is raising quickly, which can be partly due to higher medical awareness and refined identification methods (Martín-Rodríguez et al., 2017). Recent human microbiome studies have reported the presence of *Shewanella* in association with disease (Flemer et al., 2017; Kalyana Chakravarthy et al., 2018).

The genus *Shewanella* is renowned for a series of outstanding physiological and metabolic features, including an array of anaerobic respiration pathways and extracellular electron transfer mechanisms (Hau and Gralnick, 2007). Nevertheless, the genomic diversity of the genus remains obscure. One reason is the few genome sequences available in comparison to other bacterial genera (only 147 were deposited in the GenBank as per April 30th, 2019). Another reason is the rapid growth of the genus *Shewanella*, which has expanded from less than 20 species in 2003 to 67 species at present as per the List of Species with Standing Nomenclature (Parte, 2018), which still needs to be updated with some recent descriptions [e.g., *Shewanella carassii* (Fang et al., 2017)]. Some of these recently described species are based on a single isolate meeting the classic ‘gold standards’ for species definition (Moore et al., 1987; Stackebrandt et al., 2002), namely 16S rRNA sequence relatedness of ≤98.8% (Meier-Kolthoff et al., 2013b) and experimental DNA-DNA hybridization (DDH) values ≤70% as thresholds (Fournier et al., 2015; Fang et al., 2017), being otherwise metabolically and phenotypically similar to previously described species (Kim et al., 2007, 2011).

Since the first isolation of *Shewanella putrefaciens* from rotten butter in 1931 (back then *Achromobacter putrefaciens*), the taxonomy of *Shewanella* has been revisited several times (Derby and Hammer, 1931; Shewan et al., 1960; Baumann et al., 1972; MacDonell and Colwell, 1985; Ivanova et al., 2004). The first genome sequence came in 2002 for *Shewanella oneidensis* MR-1, a versatile isolate capable of using a plethora of compounds as respiratory terminal electron acceptors, including heavy metals (Heidelberg et al., 2002). It has only been relatively recently that the advent of next-generation sequencing has revolutionized our accessibility to genomic information. Overall genome related indices (OGRI) inferred from bacterial whole genome sequencing (WGS) data, such as various implementations of “Average Nucleotide Identity” (“ANI”) and digital DDH (dDDH), constitute modern tools to replace tedious traditional methods (Chun et al., 2018). But to maintain consistency in prokaryotic species delineation any novel computational approach has to be benchmarked against empirical DDH (Stackebrandt et al., 2002). In empirical comparisons of dDDH with other *in silico* measures such as “ANI,” dDDH yielded the highest correlations with traditional DDH, thus ensuring the highest consistency regarding the species-delimitation approach that currently dominates in microbial taxonomy, without sharing the disadvantages of traditional DDH (Auch et al., 2010; Meier-Kolthoff et al., 2013a). This is crucial because approaches such as “ANI” have solely been justified by their correlation with traditional DDH values, if at all (Meier-Kolthoff and Göker, 2019).

Focusing specifically on *S. algae*, we recently re-visited the taxonomic position of 33 isolates at the species level based on MLST (Martín-Rodríguez et al., 2019). We showed that species misidentification was relatively frequent within the *S. algae* clade, and we reported the first case of human infection by *S. chilikensis* upon re-classification of a clinical isolate (Martín-Rodríguez et al., 2019). The results from this first analysis involving a limited number of strains already evidenced the need for a more thorough taxonomic revision beyond the phylogenetic limits of the *S. algae* clade. To expand our analysis, in this work we have revisited the taxonomy of the genus *Shewanella* at the whole-genome level, making use of all of the WGS available for *Shewanella* spp. in public repositories.

## Materials and Methods

### Comparative Genomics of *Shewanella* Genomes

As a comparison dataset all 147 publicly available draft and complete genomes within the genus *Shewanella* as of 30-04-2019 were downloaded from GenBank. Prior to further analyses we excluded the draft genomes originating from metagenomic assemblies (*n* = 15), as well as that of *S. corallii* A687 (GCA_003353085.1), due to the presence of a *Bacillus* 16S rRNA sequence in the assembly, which suggested contamination. This resulted in a final selection of 131 *Shewanella* genomes. A complete list including accession numbers and genome characteristics is presented in Supplementary File S1, and the associated metadata are compiled in Supplementary File S2. Two genome sequences were available for the type strains of *S. putrefaciens* (JCM-20190^*T*^/GCA_000615005.1 and NBRC-3908^*T*^/GCA_001591325.1), and *S. algae* (JCM-21037^*T*^/GCA_000615045.1 and NBRC-103173^*T*^/GCA_00 1598875.1). Likewise, *S. xiamenensis* strain T17 had two genome sequences available (GCA_001723195.1 and GCA_002074855.1). Since these sequences were all submitted as separate sequencing projects we decided to include all of them in our analyses.

### Species Circumscription and Phylogeny

To study the genetic variability between the 131 selected *Shewanella* genomes, several analyses were performed as detailed below.

### Digital DNA–DNA Hybridization

All pairs of strains were compared using the Genome-to-Genome Distance Calculator 2.1 (GGDC)^1^ under recommended settings (Meier-Kolthoff et al., 2013a) and pairwise digital DNA:DNA hybridization values (dDDH) were inferred accordingly. The resulting distance matrix was subjected to a clustering using established thresholds for delineating species (DDH > 70%) (Meier-Kolthoff et al., 2013a) as well as subspecies (DDH > 79%) (Meier-Kolthoff et al., 2014b). Clustering was done using the OPTSIL clustering program (Göker et al., 2009) as previously explained (Meier-Kolthoff et al., 2014b; Liu et al., 2015).

A phylogenomic analysis based on the whole nucleotide sequences was conducted using the latest version of the Genome-BLAST Distance Phylogeny (GBDP) method (Meier-Kolthoff et al., 2013a) as previously described (Meier-Kolthoff et al., 2014a). Briefly, BLAST + (Camacho et al., 2009) was used as a local alignment tool and distance calculations were done under recommended GGDC 2.1 settings, except the use of the greedy-with-trimming algorithm and formula *d*_5_ which are better suited for phylogenetic inference (Meier-Kolthoff et al., 2014a). One hundred pseudo-bootstrap replicates were assessed under the same settings each. Finally, a balanced minimum evolution tree was inferred using FastME v2.1.4 with SPR postprocessing (Lefort et al., 2015). Replicate trees were reconstructed in the same way and branch support was subsequently mapped onto the tree.

### Average Nucleotide Identity

Average nucleotide identity (ANI) analysis (Richter and Rosselló-Móra, 2009) was performed both based on BLAST (ANIb) and MUMmer (ANIm) alignment, respectively, implemented in the *pyani* software v.0.2.7^2^.

### 16S rRNA Sequence Similarity

16S rRNA sequences from *Shewanella* type strains were retrieved from the NCBI. Pairwise sequence similarities were calculated using the method recommended by Meier-Kolthoff et al. (2013a) for the 16S rRNA gene available via the GGDC web server at https://ggdc.dsmz.de/phylogeny-service.php.

### Core Genome Comparison

To compare the genetic content of the isolates, annotation was performed using the Prokka annotation pipeline v 1.12 (Seemann, 2014) and used in the Roary pan-genome pipeline (Page et al., 2015) with an identity cut-off of 50% on protein level and the “don’t split paralogs” option to allow for differences in synteny between the species. The resulting core genome alignments were used to construct phylogenetic trees using PhyML v 3.1 (Guindon et al., 2010). Branch support was calculated using the Shimodaira-Hasegawa approximate likelihood ratio test (SH-aLRT).

### Accessory Genome Analysis

For the accessory genome comparison between the clades of contrasting G+C content we used the Roary pipeline with the more relaxed amino acid identity cut-off 50%. The presence/absence of genes in the resulting accessory genome was clustered using the pvclust package (Suzuki and Shimodaira, 2015). After verifying the divergence of the genomes with different G+C proportions, we applied the *query_pan_genome* function to identify genes unique to the clade with a high G+C content. To further aid the functional interpretation we mapped all genes unique to the clade with a high G+C content against the COG database, the KEGG database using the BlastKOALA online tool (Kanehisa et al., 2016), the Pfam protein family database (El-Gebali et al., 2019), and the NCBI Conserved Domain Database (Marchler-Bauer et al., 2017).

## Results

### Phylogenomic Analyses

We initiated our study by retrieving the 147 WGS available at the GenBank for *Shewanella*. Of note, only 34 *Shewanella* type strains had a WGS deposited. The genome sizes ranged from 2,296,595 bp (*S. putrefaciens* HRCR-6) to 6,353,406 bp (*S. psychrophila* WP2), with a G+C percentage between 39.6% (*Shewanella* sp. OPT22) and 54.8% (*Shewanella* sp. TH2012) (Supplementary File S1). After the exclusion of sequences originating from metagenomic assemblies and one with suspected contamination, our dataset consisted of 131 genomes from 128 strains (the type strains of *Shewanella algae*; [JCM-21037/NBRC-103173], *Shewanella putrefaciens*; [JCM-20190/NBRC-3908], and *Shewanella xiamenensis* T17 all had two assemblies reported, respectively).

**FIGURE 1:**
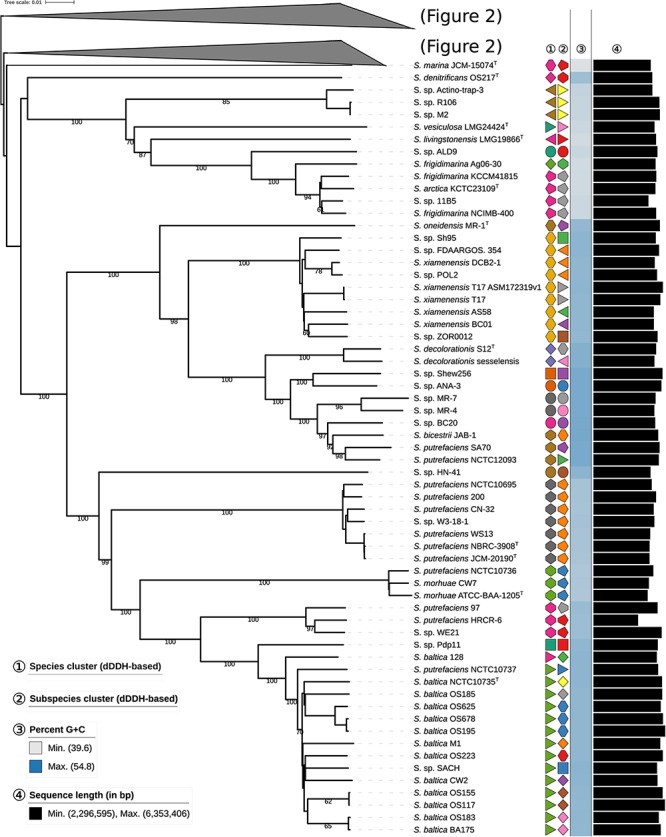
First part of the phylogenomic tree of the genus *Shewanella* inferred with GBDP. The numbers above branches are pseudo-bootstrap support values from 100 replications. Type species are indicated with a superscripted T. The two colored symbols on the right to each name refer to the species and subspecies, respectively, as determined by dDDH cutoff of 70 and 79%, respectively. The blue gradient toward the far right indicates the G+C content as calculated from WGS, followed by black bars representing the approximate genome size in bp. See the embedded legend for more details. A part of the tree has been collapsed for clarity, and is expanded in Figure 2.

**FIGURE 2:**
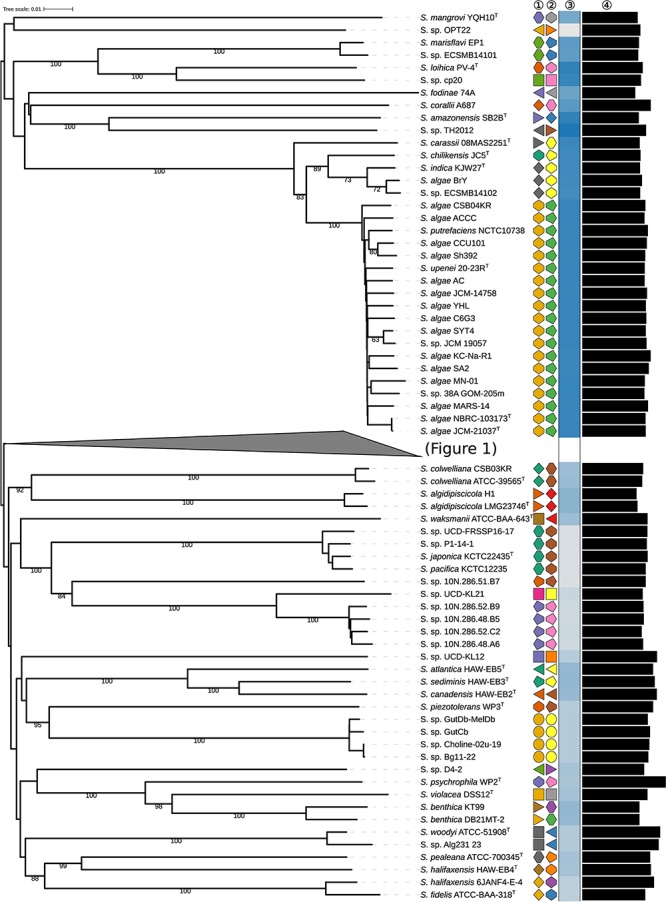
Phylogenomic tree of the genus *Shewanella* inferred with GBDP. The numbers above branches are pseudo-bootstrap support values from 100 replications. Type species are indicated with a superscripted T. The two colored symbols on the right to each name refer to the species and subspecies, respectively, as determined by dDDH cutoff of 70 and 79%, respectively. The blue gradient toward the far right indicates the G+C content as calculated from WGS, followed by black bars representing the approximate genome size in bp. See the embedded legend for more details. The part of the tree represented in Figure 1 has been collapsed for clarity.

To quantitatively assess the genomic diversity within *Shewanella*, we used dDDH (Meier-Kolthoff et al., 2014b), which enables the robust determination of both species (70% cutoff) and subspecies (79% cutoff), to reconstruct the phylogenomic associations among *Shewanella* strains with unprecedented resolution. Thus, phylogenomic relationships based on the whole nucleotide sequences were reconstructed using the GBDP method as described previously (Meier-Kolthoff et al., 2013a, 2014a) including the inference of a balanced minimum evolution tree (Lefort et al., 2015). Pairwise dDDH distances are provided in the Supplementary File S3. The resulting phylogenetic reconstruction (Figures 1, 2) revealed two major species clusters that can be readily differentiated by their G+C content. *S. loihica*, *S. amazonensis*, *S. algae*, *S. carassii*, *S. chilikensis*, and *S. indica*, with a high G+C content (48.1–53.7%; median = 53.0%) and a median genome size similar to other *Shewanella* spp. (4.31–5.20 Mbp; median = 4.80 Mbp) represent the first group. The second group comprises the remaining analyzed *Shewanella* species, which includes strains with a lower G+C proportion (39.6–50.0%; median = 45.3%) and by comparison to the first group, possessing genomes with a more diverse size range but a similar median (3.63–6.35 Mbp; median = 4.90 Mbp). Because of the scarcity of *Shewanella* genome sequences, most subclades within these major ones have few representatives. Among the most ‘populated’ ones, it is worth mentioning the heterogeneous nature of the *S. baltica* clade, comprising 13 genomes including 10 different subspecies, in contrast with, for example, the *S. putrefaciens* clade and the *S. algae* clade which are rather homogeneous even at the subspecies level.

From a taxonomic perspective, our phylogenomic reconstruction supported species (re-) assignment for 17 *Shewanella* isolates, including the consideration of *S. upenei* 20-23R Kim et al. (2011) as a heterotypic synonym of *S. algae* Simidu et al. (1990), *S. arctica* Kim et al. (2012) as a later heterotypic synonym of *S. frigidimarina* Bozal et al. (2002), and *S. pacifica* Ivanova et al. (2004) as a later heterotypic synonym of *S. japonica* Ivanova et al. (2001) (Table 1). These re-assignments were indeed also supported by ANIm inference using a 95% threshold for species designation (Table 1), with ANIm resulting otherwise in similar species clusters than dDDH (Supplementary Figure S1 and Supplementary File S4).

**TABLE 1:** Species re-assignations as determined by dDDH and ANIm.

**Strain**	**dDDH (%)**	**ANIm (%)**	**Species re-assignation**
*S. putrefaciens* NCTC10736	87.3	98.6	*S. morhuae*
*S. putrefaciens* NCTC10737	70.5	96.7	*S. baltica*
*Shewanella* sp. 38A_GOM-205 m	82.9	98.1	*S. algae*
*S. upenei* 20-23R^*T*^	83.7	98.2	*S. algae*
*Shewanella* sp. UCD-FRSSP16-17	81.7	98.1	*S. japonica*
*S. putrefaciens* NCTC10738	83.7	98.2	*S. algae*
*Shewanella* sp. JCM 19057	84.1	98.2	*S. algae*
*Shewanella* sp. P1-14-1	87.4	98.6	*S. japonica*
*S. algae* BrY	82.1	98.0	*S. indica*
*Shewanella* sp. W3-18-1	88.7	98.8	*S. putrefaciens*
*Shewanella* sp. Alg231_23	81.2	98.0	*S. woodyi*
*Shewanella* sp. ECSMB14102	82.2	98.0	*S. indica*
*Shewanella* sp. SACH	73.0	97.0	*S. baltica*
*S. halifaxensis* 6JANF4-E-4	75.2	97.4	*S. fidelis*
*S. arctica* KCTC 23109^*T*^	81.9	98.0	*S. frigidimarina*
*Shewanella* sp. 11B5	84.6	98.3	*S. frigidimarina*
*S. pacifica* KCTC12235^*T*^	88.9	98.8	*S. japonica*

Because of the lack of sufficient WGS data from *Shewanella* type strains, 47 of the 131 analyzed genomes could not be confirmed or re-defined at the species and subspecies level based on dDDH or ANIm (Supplementary Table S1). In an attempt to provide a tentative classification for these isolates, we calculated the 16S rRNA genetic distances by comparing all of the 16S rRNA sequences -including multiple copies of the gene- of the *Shewanella* type strains against each other. Our analysis (Supplementary File S5) demonstrated that 16S rRNA sequence similarity does not provide sufficient resolution at the species level within the genus *Shewanella*, with multiple type strains showing sequence similarities higher than 98.8% (Meier-Kolthoff et al., 2013b) against each other. Therefore, additional support to species designation could not be given because of the poor resolution of this marker to define species borders. However, there are several conclusions that could still be deduced for this set of 44 strains based on dDDH, as presented in Supplementary Table S1. This includes isolates whose species designation had been unambiguously misassigned (*S. putrefaciens* SA70, *S. putrefaciens* 97 and *S. baltica* 128), as well as obvious discrepancies between isolates assigned to the same species but actually representing different ones, for example *S. frigidimarina* Ag06-30 and *S. frigidimarina* NCIMB-400. The exact taxonomic position of these 44 isolates should be revisited when WGS data for type strains become available, as some might represent potentially new species.

**FIGURE 3:**
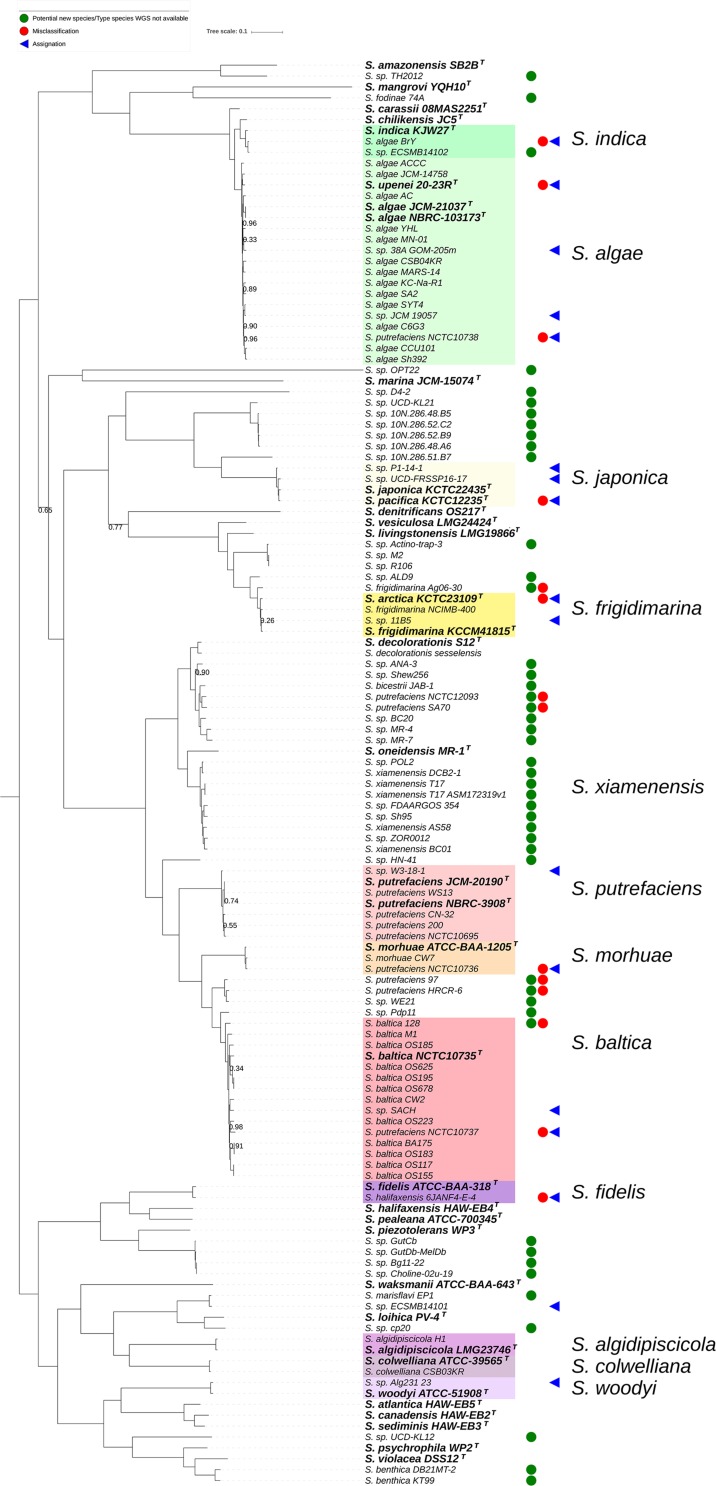
Core genome-based phylogenetic reconstruction of the genus *Shewanella* based on 131 publicly available whole genome sequences. Node figures indicate branch support values as determined by the Shimodaira-Hasegawa approximate likelihood ratio test (SH-aLRT). For clarity, no value is presented at nodes where SH-aLRT equals 1. Type species are indicated in bold with a superscripted T, and the major clades containing a type species and at least one additional genome are shadowed. A symbol has been assigned to highlight misclassified isolates (red circles), re-assignations at the species level (blue triangles) and potentially new species (green circles) based on the dDDH-based phylogeny presented in Figure 1.

To further analyze the genomic diversity across the sequenced *Shewanella* strains, we next reconstructed the phylogenomic relationships based on a core genome of 1378 protein-coding genes as determined by a 50% identity cut-off using the Roary pan-genome pipeline. Overall, the resulting approximately maximum-likelihood phylogenomic tree presented a similar topology as that of the dDDH-based tree, with minor rearrangements (Figure 3). This core genome phylogeny revealed a series well-defined major clades significantly supported as determined by the (SH-aLRT = 1). Of these, 11 contained a type species and at least one additional genome, which appear shadowed in our phylogenetic reconstruction and named after the corresponding type species (Figure 3). Several other clades as well as sub-clades within the above-mentioned displayed, however, weaker associations and therefore did not provide sufficient resolution (SH-aLRT < 0.95), most likely because of high protein sequence similarity in the core genomes involved.

**FIGURE 4:**
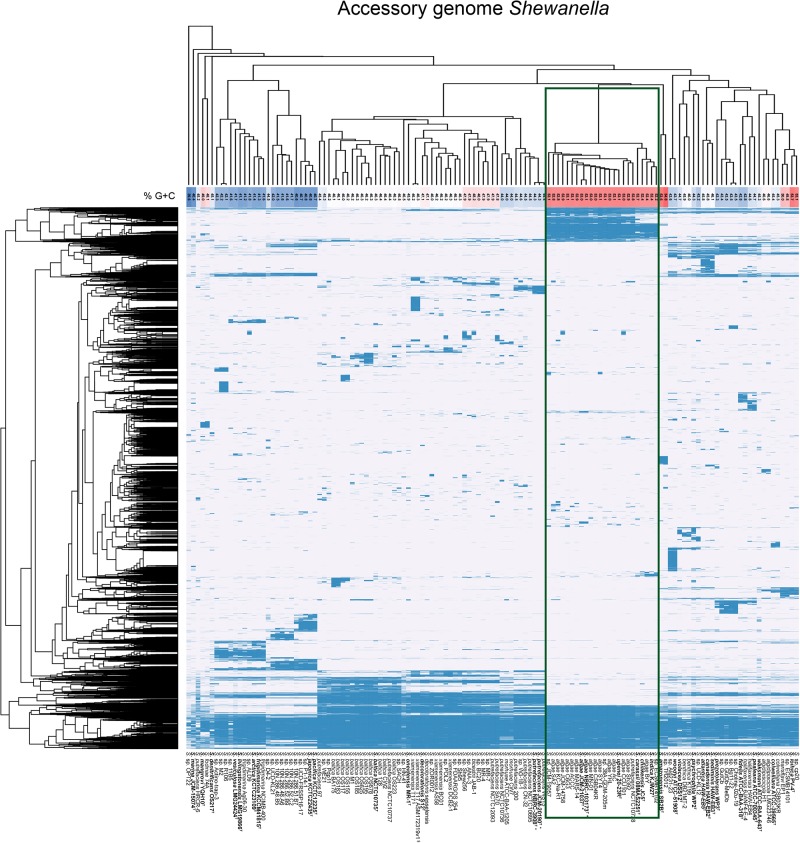
Accessory genome diversity of *Shewanella* spp. forwhich a WGS is available. Hierarchical clustering of strains and genes based on gene presence (blue) and absence (white) of the *Shewanella* accessory genes (genes present in <99% of the 131 genomes). The G+C content (%) is shaded from highest (red) to lowest (blue). The green rectangle highlights the high G+C group analyzed for unique genes. The type strains appear in bold letters with a superscripted T552269521552269521, and the two WGS sequences for the *S. putrefaciens* type strain is marked with “^+^”, the two sequences for the *S. algae* type strain is marked with “^∗^” and the two sequences for *S. xiamenensis* T17 are marked with “^§^”.

To analyze the genomic differences of the two groups of contrasting G+C proportions we performed an analysis of the accessory genomes of the strains in the dataset. The analysis with a protein identity cut-off of 50% resulted in 1378 core genes (present in >99% of the 131 genomes included in the analysis) and 36576 total gene clusters (present in between 1 and all of the genomes). When clustering the strains based on accessory (non-core) genome content the majority of the high G+C genomes grouped together (Figure 4), comprising *S. algae*, *S. chilikensis*, *S. indica*, and *S. carassii*. Exploring the gene set that was exclusive for this clade, 173 gene clusters were found to be unique for and present in all of the 22 strains in this high G+C clade (Supplementary File S6), and a total 2756 clusters were present only in genomes of this clade but none of the other genomes (data not shown), thereby indicating substantial genomic differences with respect to other *Shewanella* spp. The annotation of these clade-specific genes remains sparse since 75 of the 173 unique core genes were only annotated as being hypothetical proteins despite being mapped using COG and KEGG orthologies as well as against Pfam protein families and the Conserved Domain Database. For the genes with more detailed functional information several were involved in lipid transport and metabolism, iron and copper sequestration and transport and tryptophan degradation via the kynurenine pathway (Supplementary File S6), suggesting remarkable metabolic differences.

## Discussion

The irruption and progressive improvement of next generation sequencing (NGS) platforms such as those provided by Illumina, Pacific Biosciences and Ion Torrent, as well as improved assembly algorithms, has revolutionized the feasibility of obtaining prokaryotic genome sequence data meeting the general standards at a relatively low cost (Quail et al., 2012), prompting us in a post-genomic era. Thus, the number of bacterial genome sequences in public repositories has grown exponentially over the last decade. This provides an enormous amount of information that can be used to infer prokaryotic taxonomic relationships to an unprecedented extent based on *in silico* data, in contrast with traditional experimental approaches.

Here, we have highlighted the limitations of 16S rRNA sequence relatedness to define species borders within the genus *Shewanella*, which has been profusely reported for several bacterial taxa (Fox et al., 1992; Janda and Abbott, 2007; Fournier et al., 2015). Also, the complete *Shewanella* whole-genome sequences contained up to 14 16S rRNA sequences (*S. violacea* DSS12) between which there were slight sequence variations. Nowadays, the use of overall genome relatedness indices (OGRI) to mimic the archetypal ‘gold standard,’ conventional DDH, have been proposed as more robust, objective measures of genetic similarity (Meier-Kolthoff et al., 2013b; Chun et al., 2018). A modern replacement for the conventional DDH is required because of the laborious and error-prone nature of experimental DDH determinations which can only be performed in a few specialized laboratories worldwide. Among the several computational methods for genome-based species delineation (ANIm, ANIb, dDDH, etc.), dDDH provided the highest correlations to conventional DDH without mimicking its pitfalls (Auch et al., 2010; Meier-Kolthoff et al., 2013a; Meier-Kolthoff and Göker, 2019).

In this study, we have revisited *Shewanella* taxonomy following the standards proposed by Trujillo and co-workers (Chun et al., 2018) using dDDH to support species and subspecies designations. Thus, we were able to reassign the species of 13 genomes, with 43 additional deposited genomes whose proper species assignation needs to be revisited as new WGS from *Shewanella* type strains become available. This represents 50% of the publicly available *Shewanella* genome sequences resulting in a revised classification. The complexity of the taxonomic relationships within the genus *Shewanella*, especially within the *S. algae* clade, has been the subject of recent work by us and others (Szeinbaum et al., 2018; Martín-Rodríguez et al., 2019). Our expanded analysis reported herein resolves the taxonomy of the *S. algae* clade, which constitutes a monophyletic group distant from *S. chilikensis* and *S. indica*, and integrated by a single species, *S. algae*. Indeed, the status of *S. upenei*, which clusters within the *S. algae* clade, as a distinct species is not supported. In contrast with the experimental DDH value of 54% with *S. algae* reported in the description of this species (Kim et al., 2011), our dDDH estimates indicated 83.7%, well above the 70% threshold for species definition. Of note, *S. upenei* was also shown to produce acid from D-glucose, which has been demonstrated to be a variable trait in *S. algae* (Satomi, 2014). Such phenotypic characteristics, while helpful to discriminate between closely related species routinely, should not be regarded as conclusive because of the multiple biotypes that might exist within any given species. Based on the rules of priority reflected in the International Code of Nomenclature of Prokaryotes (Parker et al., 2019), *S. upenei* Kim et al. (2011) should be regarded as a later heterotypic synonym of *S. algae* Simidu et al. (1990), as recently reported for *S. haliotis* Kim et al. (2007) (Szeinbaum et al., 2018). We also showed that *Shewanella* sp. ECSMB14102 and *S. algae* BrY, whose potential entity as a first member of a new *Shewanella* species had been proposed (Szeinbaum et al., 2018), are actually *S. indica* as determined by dDDH and ANIm.

The maximally supported *S. algae* subtree (Figure 2) is located in a larger, G+C-rich subtree comprising *S. indica*, *S. chilikensis* and *S. carassii*, which constitutes an expansion of what was formerly called Owen’s group IV (Owen et al., 1978). As demonstrated by our accessory genome analysis, this G+C-rich subtree exhibits substantial differences in genomic content with respect to other phylogroups within the genus. The annotation of *Shewanella* genomes is still poor, with a high proportion of hypothetical genes whose function or involvement in certain physiological processes is still unknown. Despite this, we could identify a series of genes that could be potentially associated to some typical features of this subtree, including, for example, tryptophan degradation via the kynurenine pathway and iron sequestration, potentially related to melanin-mediated iron acquisition (Turick et al., 2002) and pigmentation on L-tryptophan agar (Khashe and Janda, 1998). Interestingly, among the unique genes for G+C-rich *Shewanella* spp. are the *avb* and *avt* genes involved in the biosynthesis of the siderophore avaroferrin (Böttcher and Clardy, 2014; Rütschlin et al., 2017).

## Conclusion

In conclusion, in this work we have provided an up-to-date taxonomic revision of the genus *Shewanella* upon analysis of the genome sequences publicly available, providing for the first time a whole genome-based taxonomy for this genus at the species and sub-species level. The taxonomic position of almost half of the analyzed genomes has been reassigned or needs to be revisited when additional genomic data become available. Three type strains, *S. upenei*, *S. arctica* and *S. pacifica*, have been demonstrated to be later heterotypic synonyms of previously described species. A major taxonomic limitation to shed further light on the taxonomy of this genus is the scarcity of *Shewanella* type-strain genome sequences. Furthermore, with only 131 sequences available representing less than 50% of the *Shewanella* species, our study emphasizes the need of collective efforts to increase our genomic understanding of this genus, which will redound in improved knowledge on its physiology, ecology and evolution. Improved gene annotation and experimental validation studies are certainly needed to overcome the tremendous gap that still exists in this aspect. Further taxonomic updates will be certainly needed in the coming years.

### Taxonomic Consequences

#### Redefinition of *S. upenei* Kim et al. (2011) as a Later Heterotypic Synonym of *S. algae* Simidu et al. (1990)

The status of *S. upenei* (Kim et al., 2011) as a species is not supported by both ANIm and dDDH, clustering unambiguously within the *S. algae* clade. Therefore, in accordance with the Rule for Prokaryotic Nomenclature 24b (Parker et al., 2019), *S. upenei* should be considered a later heterotypic synonym of *S. algae*.

#### Redefinition of *S. arctica* Kim et al. (2012) as a Later Heterotypic Synonym of *S. frigidimarina* Bozal et al. (2002)

The status of *S. arctica* (Kim et al., 2012) as a species is not supported by both ANIm and dDDH. In accordance with the Rule for Prokaryotic Nomenclature 24b (Parker et al., 2019), *S. pacifica* should be considered a later heterotypic synonym of *S. frigidimarina*.

#### Redefinition of *S. pacifica* Ivanova et al. (2004) as a Later Heterotypic Synonym of *S. japonica* Ivanova et al. (2001)

The status of *S. pacific* (Ivanova et al., 2004) as a species is not supported by both ANIm and dDDH. In accordance with the Rule for Prokaryotic Nomenclature 24b (Parker et al., 2019), *S. pacifica* should be considered a later heterotypic synonym of *S. japonica*.

#### Emended Description of *Shewanella algae* Simidu et al. (1990) Emend. Nozue et al. (1992)

The description is as before (Simidu et al., 1990; Nozue et al., 1992) with the following modification: the G+C content of the type-strain genome (OK-1 = IAM 14159 = ATCC 51192 = CCUG 39064 = CIP 106454 = CECT 5071 = JCM 21037 = NBRC 103173) is 53.0% (range for sequenced strains: 52.4–53.1%), its approximate size is 4.83 Mbp (range for sequenced strains: 4.55–5.20 Mbp), and its GenBank accession is GCA_000615045.1/GCA_001598875.

#### Emended Description of *Shewanella algidipiscicola* Satomi et al. (2007)

The description is as before (Satomi et al., 2007) with the following modification: the G+C content of the type-strain (S13 = LMG 23746 = NBRC 102032) is 46.6%, (range for sequenced strains: 46.6–46.6%), its approximate size is 4.20 Mbp (range for sequenced strains: 4.14–4.20 Mbp), and its GenBank accession is GCA_003605125.1.

#### Emended Description of *Shewanella amazonensis* Venkateswaran et al. (1998)

The description is as before (Venkateswaran et al., 1998) with the following modification: the G+C content of the type-strain (SB2B = ATCC 700329 = CIP 105786) genome is 53.6%, its approximate size is 4.31 Mbp, and its GenBank accession is GCA_000015245.1.

#### Emended Description of *Shewanella atlantica* Zhao et al. (2007)

The description is as before (Zhao et al., 2007) with the following modification: the G+C content of the type-strain (HAW-EB5 = CCUG 54554 = NCIMB 14239) genome is 45.8%, its approximate size is 5.39 Mbp, and its GenBank accession is GCA_003966265.1.

#### Emended Description of *Shewanella baltica* Ziemke et al. (1998)

The description is as before (Ziemke et al., 1998) with the following modification: the G+C content of the type-strain (CCUG 39356 = CECT 323 = CIP 105850 = DSM 9439 = IAM 1477 = LMG 2250 = NCTC 10735) genome is 46.3% (range for sequenced strains: 46.0–46.4%), its approximate size is 5.30 Mbp (range for sequenced strains: 4.90–5.55 Mbp), and its GenBank accession is GCA_900456975.1.

#### Emended Description of *Shewanella canadensis* Zhao et al. (2007)

The description is as before (Zhao et al., 2007) with the following modification: the G+C content of the type-strain (HAW-EB2 = CCUG 54553 = NCIMB 14238) genome is 45.8%, its approximate size is 5.68 Mbp, and its GenBank accession is GCA_003966225.1.

#### Emended Description of *Shewanella chilikensis* Sucharita et al. (2009)

The description is as before (Sucharita et al., 2009) with the following modification: the G+C content of the type-strain (JC5 = CCUG 57101 = KCTC 22540 = NBRC 105217) genome is 52.4%, its approximate size is 4.44 Mbp, and its GenBank accession is GCA_003217175.1.

#### Emended Description of *Shewanella colwelliana* Coyne et al. (1989)

The description is as before (Coyne et al., 1989) with the following modification: the G+C content of the type-strain (LST-W = ATCC 39565) genome is 45.4% (range for sequenced strains: 45.3–45.4%), its approximate size is 4.58 Mbp (range for sequenced strains: 4.58–4.64 Mbp), and its GenBank accession is GCA_000518705.

#### Emended Description of *Shewanella decolorationis* Xu et al. (2005)

The description is as before (Xu et al., 2005) with the following modification: the G+C content of the type-strain (S12 = CCTCC M 203093 = IAM 15094 = JCM 21555 = NBRC 103170) genome is 47.1% (range for sequenced strains: 47.1–47.1%), its approximate size is 4.84 Mbp (range for sequenced strains: 4.72–4.84 Mbp), and its GenBank accession is GCA_000485795.1.

#### Emended Description of *Shewanella denitrificans* Brettar et al. (2002)

The description is as before (Brettar et al., 2002) with the following modification: the G+C content of the type-strain (OS217 = DSM 15013 = LMG 21692) genome is 45.1%, its approximate size is 4.55 Mbp, and its GenBank accession is GCA_000013765.1.

#### Emended Description of *Shewanella fidelis* Ivanova et al. (2003b)

The description is as before (Ivanova et al., 2003b) with the following modification: the G+C content of the type-strain (ATCC BAA-318 = KMM 3582 = LMG 20552) genome is 42.8%, its approximate size is 4.80 Mbp, and its GenBank accession is GCA_000518605.1.

#### Emended Description of *Shewanella frigidimarina* Bozal et al. (2002)

The description is as before (Bozal et al., 2002) with the following modification: the G+C content of the type-strain (ACAM 591 = ATCC 700753 = CIP 105515) genome is 40.7%, its approximate size is 4.83 Mbp, and its GenBank accession is GCA_003605145.1.

#### Emended Description of *Shewanella halifaxensis* Zhao et al. (2006)

The description is as before (Zhao et al., 2006) with the following modification: the G+C content of the type-strain (HAW-EB4 = DSM 17350 = NCIMB 14093) genome is 44.6% (range for sequenced strains: 42.8–44.6%), its approximate size is 5.23 Mbp (range for sequenced strains: 5.23–5.46 Mbp), and its GenBank accession is GCA_000019185.1.

#### Emended Description of *Shewanella indica* Verma et al. (2011)

The description is as before (Verma et al., 2011) with the following modification: the G+C content of the type-strain (KJW27 = BCC 41031 = KCTC 23171 = NCIM 5388) genome is 52.4%, its approximate size is 4.40 Mbp, and its GenBank accession is GCA_002836975.1.

#### Emended Description of *Shewanella japonica* Ivanova et al. (2001)

The description is as before (Ivanova et al., 2001) with the following modification: the G+C content of the type-strain (ATCC BAA-316 = CIP 106860 = JCM 21433 = KMM 3299 = LMG 19691 = NBRC 103171) genome is 40.8%, its approximate size is 4.98 Mbp, and its GenBank accession is GCA_002075795.1.

#### Emended Description of *Shewanella livingstonensis* Bozal et al. (2002)

The description is as before (Bozal et al., 2002) with the following modification: the G+C content of the type-strain (NF22 = LMG 19866 = CECT 5933) genome is 41.1%, its approximate size is 4.84 Mbp, and its GenBank accession is GCA_003855395.1.

#### Emended Description of *Shewanella loihica* Gao et al. (2006)

The description is as before (Gao et al., 2006) with the following modification: the G+C content of the type-strain (PV-4 = ATCC BAA-1088 = DSM 17748) genome is 53.7%, its approximate size is 4.60 Mbp, and its GenBank accession is GCA_000016065.1.

#### Emended Description of *Shewanella mangrovi* Liu et al. (2015)

The description is as before (Zeng et al., 2015) with the following modification: the G+C content of the type-strain (YQH10 = MCCC 1A00830 = JCM 30121) genome is 48.1%, its approximate size is 4.22 Mbp, and its GenBank accession is GCA_000753795.1.

#### Emended Description of *Shewanella marina* Park et al. (2009)

The description is as before (Park et al., 2009) with the following modification: the G+C content of the type-strain (C4 = JCM 15074 = KCTC 22185) genome is 40.4%, its approximate size is 4.42 Mbp, and its GenBank accession is GCA_000614975.1/ASM61497v1.

#### Emended Description of *Shewanella morhuae* Satomi et al. (2006)

The description is as before (Satomi et al., 2006) with the following modification: the G+C content of the type-strain (U1417 = ATCC BAA-1205 = NBRC 100978) genome is 44.0% (range for sequenced strains: 44.0–44.0%), its approximate size is 4.19 Mbp (range for sequenced strains: 4.19–4.29 Mbp), and its GenBank accession is GCA_900156405.1.

#### Emended Description of *Shewanella oneidensis* Venkateswaran et al. (1999)

The description is as before (Venkateswaran et al., 1999) with the following modification: the G+C content of the type-strain (MR-1 = ATCC 700550 = CIP 106686 = LMG 19005) genome is 45.9%, its approximate size is 5.13 Mbp, and its GenBank accession is GCA_000146165.2.

#### Emended Description of *Shewanella pealeana* Leonardo et al. (1999)

The description is as before (Leonardo et al., 1999) with the following modification: the G+C content of the type-strain (ANG-SQ1 = ATCC 700345 = CIP 106450) genome is 44.7%, its approximate size is 5.17 Mbp, and its GenBank accession is GCA_000018285.1.

#### Emended Description of *Shewanella piezotolerans* Xiao et al. (2007)

The description is as before (Xiao et al., 2007) with the following modification: the G+C content of the type-strain (WP3 = CGMCC 1.6160 = JCM 13877) genome is 43.3%, its approximate size is 5.40 Mbp, and its GenBank accession is GCA_000014885.1.

#### Emended Description of *Shewanella psychrophila* Xiao et al. (2007)

The description is as before (Xiao et al., 2007) with the following modification: the G+C content of the type-strain (WP2 = CGMCC 1.6159 = JCM 13876) genome is 44.3%, its approximate size is 6.35 Mbp, and its GenBank accession is GCA_002005305.1.

#### Emended Description of *Shewanella putrefaciens* Lee et al. (1977)

The description is as before (Lee et al., 1977) with the following modification: the G+C content of the type-strain (Hammer 95 = ATCC 8071 = CCUG 13452 D = CFBP 3033 = CFBP 3034 = CIP 80.40 = DSM 6067 = IFO (now NBRC) 3908 = JCM 9294 = JCM 20190 = LMG 2268 = NCIB (now NCIMB) 10471 = NCTC 12960) genome is 44.3% (range for sequenced strains: 44.0–45.5%), its approximate size is 4.34 Mbp (range for sequenced strains: 3.63–4.95 Mbp), and its GenBank accession is GCA_000615005.1.

#### Emended Description of *Shewanella sediminis* Zhao et al. (2005)

The description is as before (Zhao et al., 2005) with the following modification: the G+C content of the type-strain (HAW-EB3 = DSM 17055 = NCIMB 14036) genome is 46.1%, its approximate size is 5.52 Mbp, and its GenBank accession is GCA_000018025.1.

#### Emended Description of *Shewanella vesiculosa* Bozal et al. (2009)

The description is as before (Bozal et al., 2009) with the following modification: the G+C content of the type-strain (M7 = CECT 7339 = LMG 24424) genome is 41.7%, its approximate size is 4.72 Mbp and its GenBank accession is GCA_000091325.1.

#### Emended Description of *Shewanella violacea* Nogi et al. (1998)

The description is as before (Nogi et al., 1998) with the following modification: the G+C content of the type-strain (DSS12 = CIP 106290 = JCM 10179 = LMG 19151) genome is 44.7%, its approximate size is 4.96 Mbp and its GenBank accession is GCA_000091325.1/ASM9132v1.

#### Emended Description of *Shewanella waksmanii* Ivanova et al. (2003a)

The description is as before (Ivanova et al., 2003a) with the following modification: the G+C content of the type-strain (ATCC BAA-643 = CIP 107701 = KMM 3823) genome is 45.3%, its approximate size is 4.97 Mbp, and its GenBank accession is GCA_000518805.1.

#### Emended Description of *Shewanella woodyi* Makemson et al. (1997)

The description is as before (Makemson et al., 1997) with the following modification: the G+C content of the type-strain (MS32 = ATCC 51908 = CIP 105547 = DSM 12036) genome is 43.7%, its approximate size is 5.94 Mbp, and its GenBank accession is GCA_000019525.1.

## Data Availability

Publicly available datasets were analyzed in this study. This data can be found here: GCA_003024535.1, GCA_003025175.1, GCA_001870495.1, GCA_000956365.1, GCA_003427415.1, GCA_001858195.1, GCA_000614935.1, GCA_000615045.1, GCA_000947195.1, GCA_002237105.1, GCA_001598875.1, GCA_003124085.1, GCA_003024575.1, GCA_002318995.1, GCA_003721455.1, GCA_004153715.2, GCA_900380485.1, GCA_003605125.1, GCA_000015245.1, GCA_003797125.1, GCA_003966265.1, GCA_003052765.1, GCA_000147735.3, GCA_003030925.1, GCA_001620325.1, GCA_900456975.1, GCA_000215895.1, GCA_000015845.1, GCA_000179535.2, GCA_000017325.1, GCA_000018765.1, GCA_000021665.1, GCA_000231345.2, GCA_000178875.2, GCA_900476435.1, GCA_000172075.1, GCA_002216875.1, GCA_003966225.1, GCA_002777975.1, GCA_003217175.1, GCA_000518705.1, GCA_001735525.1, GCA_000485795.1, GCA_004354305.1, GCA_000013765.1, GCA_000518605.1, GCA_004342405.1, GCA_001529365.1, GCA_000014705.1, GCA_003797845.1, GCA_003112715.1, GCA_000019185.1, GCA_002836975.1, GCA_002075795.1, GCA_003855395.1, GCA_000016065.1, GCA_000753795.1, GCA_000614975.1, GCA_002215585.1, GCA_900156405.1, GCA_003028295.1, GCA_000146165.2, GCA_003605145.1, GCA_000018285.1, GCA_000014885.1, GCA_002005305.1, GCA_003315425.1, GCA_000169215.2, GCA_000016585.1, GCA_000519065.1, GCA_000615005.1, GCA_001591325.1, GCA_900457065.1, GCA_900457045.1, GCA_900457125.1, GCA_002157365.1, GCA_003044255.1, GCA_900636855.1, GCA_900636665.1, GCA_000018025.1, GCA_002873135.1, GCA_002874355.1, GCA_002873615.1, GCA_002873635.1, GCA_002874545.1, GCA_002836795.1, GCA_000518445.1, GCA_002836275.1, GCA_002836315.1, GCA_900079515.1, GCA_000203935.1, GCA_003129585.1, GCA_002836615.1, GCA_002836205.1, GCA_000832025.1, GCA_000773485.1, GCA_000813075.1, GCA_002209245.2, GCA_002836135.1, GCA_002836075.1, GCA_000217915.2, GCA_001310535.1, GCA_000014685.1, GCA_000014665.1, GCA_001401775.1, GCA_000282755.1, GCA_002838165.1, GCA_001887095.2, GCA_001308045.1, GCA_002196695.1, GCA_001675935.1, GCA_001957125.1, GCA_001957135.1, GCA_000015185.1, GCA_002966515.1, GCA_000798835.1_ZOR0012.1, GCA_003855155.1, GCA_ 004295345.1, GCA_004168585.1, GCA_003797165.1, GCA_003957745.1, GCA_002836995.1, GCA_003797885.1, GCA_000091325.1, GCA_000518805.1, GCA_000019525.1, GCA_002738015.1, GCA_000712635.2, GCA_003130545.1, GCA_001723195.1, GCA_002074855.1.

## Author Contributions

KT and JM-K performed the bioinformatic analyses. ÅS provided facilities and resources. AM-R conceived the work, guided the study, and wrote the manuscript. All authors contributed to data analysis and editing.

## Conflict of Interest Statement

The authors declare that the research was conducted in the absence of any commercial or financial relationships that could be construed as a potential conflict of interest.

## Footnotes

1 https://ggdc.dsmz.de

2 https://github.com/widdowquinn/pyani
